# Cardiac herniation post left upper lobectomy and thymectomy: a case report

**DOI:** 10.1186/s13019-024-02713-y

**Published:** 2024-04-16

**Authors:** Hiral Jhala, Mathew Thomas

**Affiliations:** Department of Cardiothoracic Surgery, Golden Jubilee National Hospital, Glasgow, G81 4DY Scotland

**Keywords:** Cardiac herniation, Left upper lobectomy, Thymectomy

## Abstract

**Background:**

Cardiac herniation occurs when there is a residual pericardial defect post thoracic surgery and is recognised as a rare but fatal complication. It confers a high mortality and requires immediate surgical correction upon recognition. We present a case of cardiac herniation occurring post thymectomy and left upper lobectomy.

**Case presentation:**

***Initial presentation***: A 48-year-old male, hypertensive smoker presented with progressive breathlessness and was found to have a left upper zone mass confirmed on CT biopsy as carcinoid of unclear origin. PET-CT revealed avidity in a left anterior mediastinal area, left upper lobe (LUL) lung mass, mediastinal lymph nodes, and a right thymic satellite nodule. ***Intraoperatively***: Access via left thoracotomy and sternotomy. The LUL tumour involved the left thymic lobe (LTL), left superior pulmonary vein (LSPV), left phrenic nerve and intervening mediastinal fat and pericardium, which were resected en-masse. The satellite nodule in the right thymic lobe (RTL) was adjacent to the junction between the left innominate vein and superior vena cava (SVC). The pericardium was resected from the SVC to the left atrial appendage. ***Clinical deterioration***: Initially the patient was doing well clinically on day 1, however there was sudden bradycardia, hypotension, clamminess, and oligoanuria, with raised central venous pressures and troponins. ECG: no capture in leads V1-2, but positive deflections seen on posterior leads. Echo: no acoustic windows, but good windows seen posteriorly. CXR: left mediastinal shift. ***Redo operation***: After initial resuscitation and stabilisation on the intensive care unit, on day 2 a redo-sternotomy revealed cardiac herniation into the left thoracic cavity with the left ventricular apex pointing towards the spine, and inferior caval kinking. After reduction and repair of the pericardial defect with a fenestrated GoreTex patch, the patient recovered well with complete resolution of the ECG and CXR.

**Conclusion:**

Cardiac herniation can even occur following sub-pneumonectomy lung resections and should be considered as a differential when faced with a sudden clinical deterioration, warranting early surgical correction.

## Background

Cardiac herniation was first reported as a complication following lung resection in 1948 involving pericardiectomy [1]. It has since been recognised as a rare but fatal complication, conferring a high mortality and requiring immediate surgical correction upon recognition. Cardiac herniation following thoracic surgery usually occurs when there is a residual pericardial defect creating an empty space in the adjoining thoracic cavity. The vast majority of cases reported in the literature have occurred following intrapericardial pneumonectomy [2, 3]. However, there is minimal literature describing cardiac herniation following lung resections smaller than pneumonectomies. We present a case of cardiac herniation occurring post thymectomy and left upper lobectomy.

## Case report

### Initial presentation

**Fig. 1:**
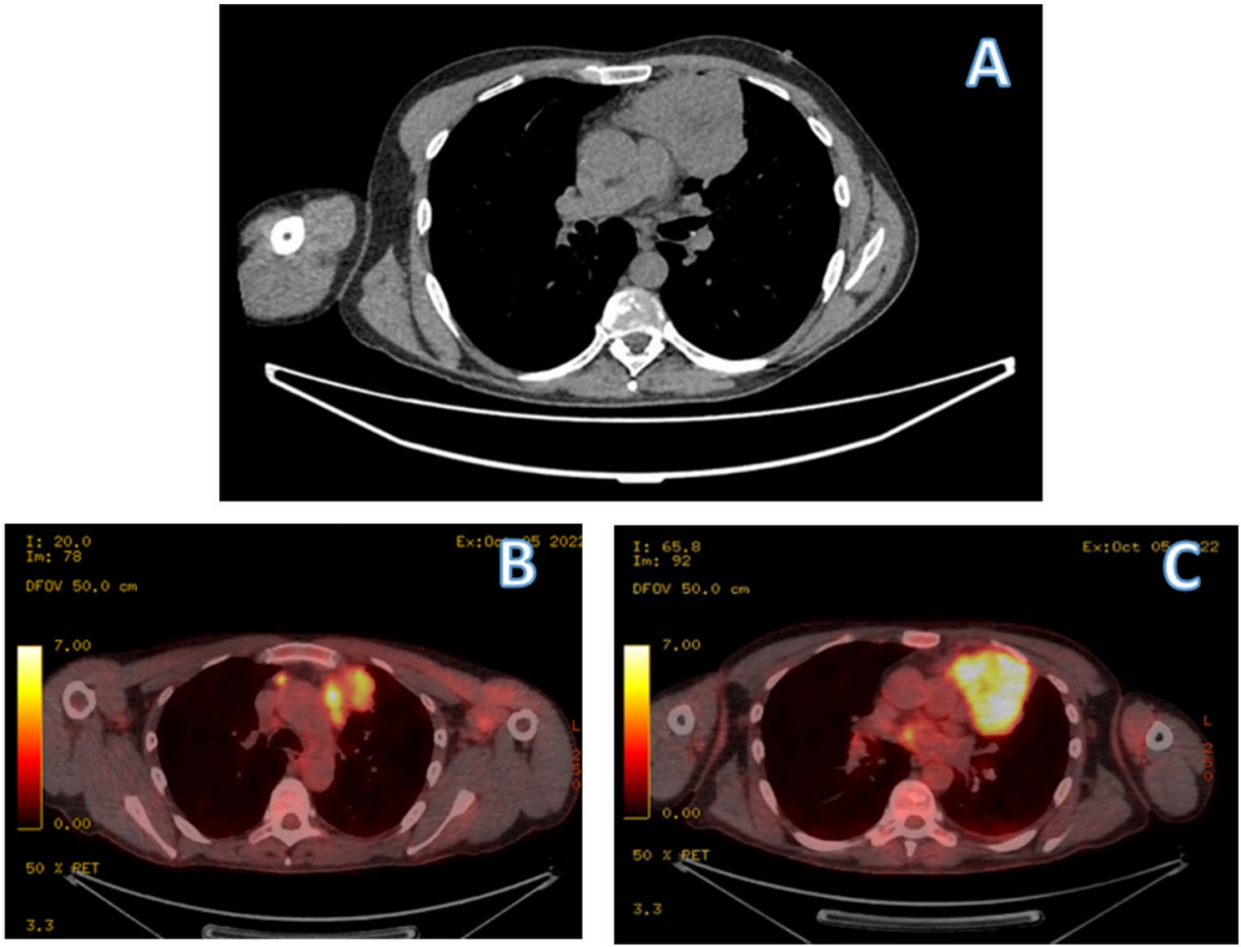
**A** Axial CT scan demonstrating left anterior mediastinal mass **B** & **C** Axial PET showing FDG avid right thymic nodule and left anterior mediastinal tumour involving the left upper lobe

A 48-year-old hypertensive smoker, presented with a history of progressive shortness of breath (Medical Research Council dyspnoea grade 2) [4]. Initial clinical examination and laboratory tests were unremarkable for thoracic pathology. A chest radiograph (CXR) identified a widened mediastinum along with a left upper zone mass. This was confirmed on subsequent Computed Tomography (CT) and Positron-Emission Tomography (PET) scans (Fig. 1a, b and c) as being an fluorodeoxyglucose (FDG) avid left anterior mediastinal and upper lobe lung mass with an avid station 5 mediastinal lymph node, and a PET avid right thymic satellite nodule seen adjacent to the junction between the left innominate vein and superior vena cava (SVC). A CT biopsy of the mediastinal mass showed carcinoid of unknown origin. This was staged as T4N3M0 [5]. T4 due to mediastinal and lung involvement and N3 due to the right thymic nodule which was debated as lymph node or nodule. Staging nodal assessment was not felt possible as only station 5 was avid and pathological. The MDT considered surgical and oncological treatment options and given the carcinoid diagnosis, surgical excision was felt to be the apt option.

### Intraoperatively

The surgical targets were the right thymic nodule, the left thymic lobe and the left upper lung lobe. To reduce the incision related morbidity, the initial plan was keyhole access for the left hemithorax and then a sternotomy to inspect the right thymic area abnormality and its possible excision. On inspection of the left hemithorax via robotic access, the tumour was found to involve the left upper lobe of the lung with direct confluent spread to the left thymic lobe and left phrenic nerve. Given the adhesions and bulk, we converted to a left posterolateral thoracotomy and completed the left upper lobectomy with complete gross excision of neighbouring mediastinal fat, left phrenic nerve and lymphadenectomy of station 5 with sampling of left stations 10, 11, 9 and 7. The main specimen was left attached to the left thymic lobe to avoid any spillage. The thoracotomy was closed, patient rolled into a supine position and a median sternotomy was performed. The right thymic nodule from the CT was found as a discrete nodule in the body of the right thymic lobe adjacent to the junction between the left brachiocephalic vein and superior vena cava (SVC). Due to dense adhesions with the pericardium, both thymic lobes were radically resected with the underlying attached pericardium. The entire detached specimen was extracted without spillage in a large retrieval bag via the sternotomy. The residual pericardial defect was from the SVC to the left atrial appendage (LAA). Although the pericardial defect was large, it was felt that this defect was mostly anterior and cardiac herniation could be prevented by left lower lobe expansion and a raised left hemidiaphragm. 2 pleural and 1 mediastinal chest drains were placed on suction.

### **Clinical deterioration**

**Fig. 2:**
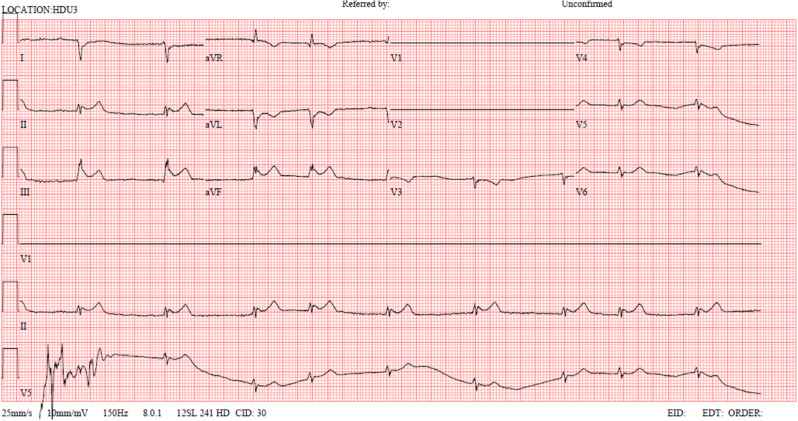
Initial 12-lead ECG of patient showing junctional rhythm and lack of capture in chest leads V1-V2

**Fig. 3:**
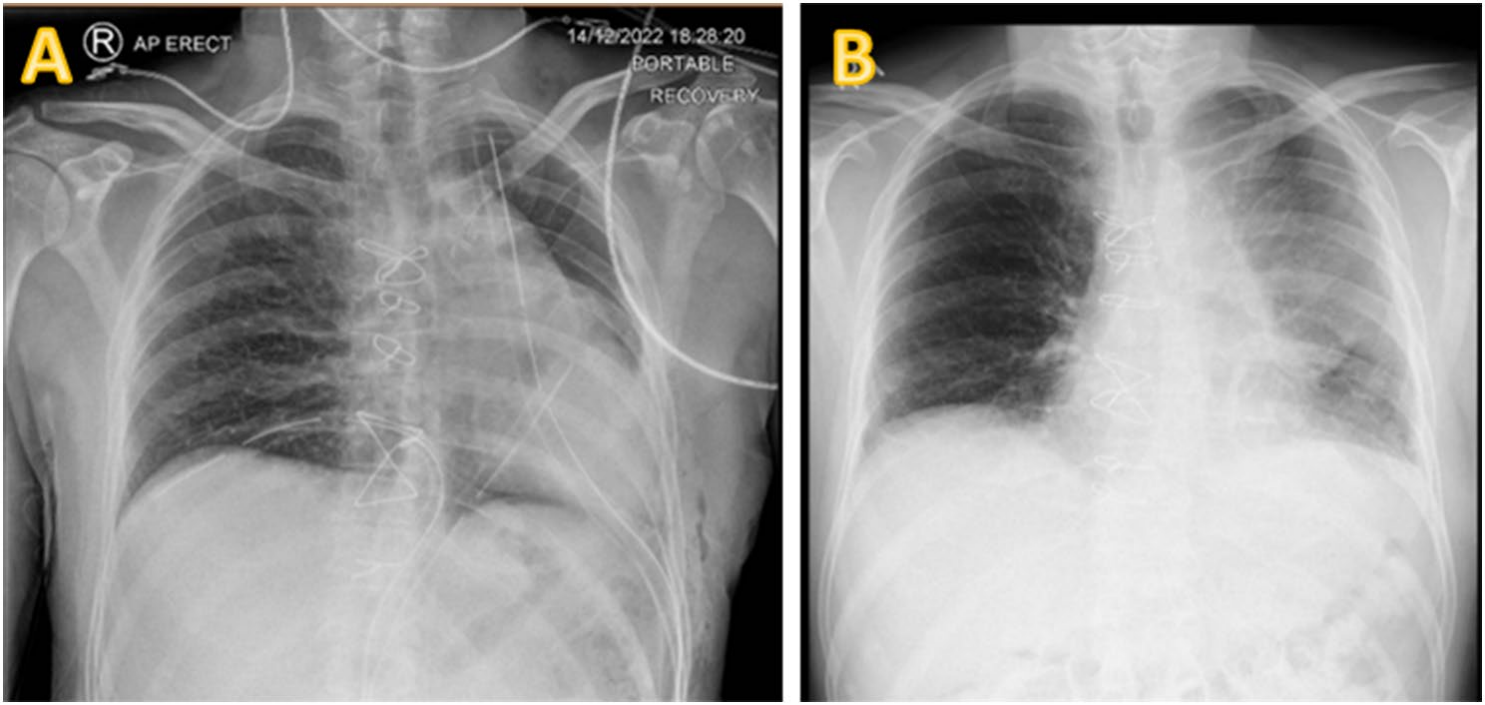
**A** CXR pre repair of cardiac herniation showing mediastinal shift **B** CXR post reduction and repair of pericardial defect, showing a midline mediastinum

On day 1 post-operatively, the patient looked well, had stable observations and was mobilising adequately. Later in the day, he suddenly became clammy with oligoanuria but normotension (103/76mmHg) and bradycardia. A newly placed central venous catheter showed a central venous pressure (13mmHg) and raised troponins (1073 ng/L). The blood pressure responded to intravenous crystalloid boluses but his 12-lead ECG showed bradycardia with a junctional rhythm, with no capture in chest leads V1-2 (Fig. 2). A bedside transthoracic echocardiogram could not obtain sufficient acoustic windows. A CXR showed left sided shift of the mediastinal contents (Fig. 3a). When posterior ECG leads were applied along the inferior scapular line, as was the echocardiogram probe, positive deflections and acoustic windows were seen. All these features together were suggestive of cardiac herniation. He was transferred to the intensive care unit for haemodynamic optimisation with judicious filling and dobutamine.

### **Redo operation**

The patient underwent a redo-sternotomy on day 2. The heart had herniated into the left thoracic cavity with the left ventricular apex pointing towards the spine, causing marked inferior caval kinking. After reducing the heart back to its normal anatomical lie, the pericardial defect (approximately 15 cm x 10 cm) was repaired using a GoreTex patch. Fenestrations were made in the patch to prevent cardiac tamponade. Bronchoscopy showed endobronchial secretions which were suctioned out. The left lower lobe re-expanded well. The post-operative CXR demonstrated resolution of the mediastinal shift and adequate left lung re-expansion (Fig. 3b**).** The ECG trace returned to normal sinus rhythm. The patient recovered well clinically and biochemically. The final histopathology showed a completely resected TTF1 negative atypical carcinoid pT3N2 (from station 5) M0 R0 of thymic origin.

## Discussion

### Recognition and diagnosis

This presentation occurred 24 h post-operatively. Acute presentations of cardiac herniation post thoracic surgery occur within the 24–48 h post-operatively, most commonly during repositioning [6]. The literature reports expected clinical features of cardiac herniation to be hypotension, tachycardia and arrhythmias along with symptoms depending on laterality (obstructed venous return on the right or ischaemic cardiac compression and low output on the left) [7]. Our patient was bradycardic with a junctional rhythm, likely due to mechanical pressure on the cavo-atrial junction caused by herniation, thus blocking sino-atrial nodal conduction but the atrio-ventricular nodal pathways remaining intact.

Cardiac herniations have been reported as presenting with inferior myocardial ischaemia [8] which although absent in our patient, could have been a consequence had there not been an early suspicion of torsion or herniation. Whilst more commonly described post intrapericardial pneumonectomy and thoracic trauma, cardiac herniation can be a complication following even minimally invasive thymectomy [9] and cardiac surgery [10]. Furthermore, it is possible that applying negative pressure drainage post lobectomy, may have exacerbated herniation, in an otherwise smaller cavity bound by a raised hemidiaphragm.

### **Intraoperative management**

In hindsight we question whether the pericardial defect should have been repaired in the first operation. Although the pericardial defect was around 10 × 15 cm, it was primarily anterior and given that the lung resection was not a pneumonectomy, it was felt that the left lower lobe and a raised left hemidiaphragm could be obstructive enough to prevent cardiac herniation. We hypothesise, that the left lower lobe did not adequately re-expand due to endobronchial secretions and perhaps incision related pain, which eventually provided enough empty space for the heart to herniate. Definitive criteria are yet to be identified for the size of pericardial defect beyond which patch repair is recommended. Some report repairs of pericardial defects of sizes of 4 cm × 4cm [11] and 5 cm × 3 cm [12]. This experience highlights that due consideration should be given to repairing reasonably large pericardial defects. Once the repair was performed, the improvement in the patient’s clinical and biochemical picture was impressive.

## Conclusion

Cardiac herniation occurs between 24 and 48 h post operatively, and should be considered as a differential when faced with a sudden clinical deterioration. Immediate surgical repair should be undertaken in a timely fashion to prevent serious arrhythmias, ischaemia or infarction and loss of output. Although thought of as a complication more common post pneumonectomy, our case highlights the possibility even after extensive thymic resection involving a single lobectomy.

## Data Availability

The datasets used and/or analysed for this case report are available from the corresponding author on reasonable request.
